# Virtual CARE for Caregivers of PWD: Adapting to the New Normal

**DOI:** 10.1093/geroni/igab046.3647

**Published:** 2021-12-17

**Authors:** Steven Shirk, Maureen O'Connor, Jaye McLaren, Kendra Pugh, Andrew Nguyen, Sarah Boucher, Lauren Moo

**Affiliations:** 1 VA Bedford Healthcare System, Pepperell, Massachusetts, United States; 2 VA Bedford Healthcare System, Bedford, Massachusetts, United States; 3 VA Bedford Healthcare System, VA Bedford Healthcare System, Massachusetts, United States; 4 Boston University, Boston, Massachusetts, United States; 5 VA Bedford Health Care System, Bedford, Massachusetts, United States

## Abstract

Caregivers of persons with dementia (PWD) often experience increases in depression, anxiety, and burden as the disease progresses. In fact, as the PWD’s neuropsychiatric symptoms increase and independence in ADLS decrease, caregivers psychological and physical health outcomes worsen. The literature suggests that caregiver interventions that teach specific skills are more beneficial than psychoeducational interventions, particularly regarding the amelioration of the psychological impacts of informal caregiving. However, because of caregiving demands, caregiver’s own physical limitations, and competing obligations, it can be difficult to attend caregiver support or education programs outside the home. With the emergence of the COVID-19 pandemic, arranging such interventions became more complex, Therefore, we report on preliminary qualitative outcomes of a study investigating the feasibility and acceptability of converting an in-person, group dementia caregiver education intervention, CARE, to a telehealth platform. We report the findings of two objectives: 1) lessons learned when attempting to convert an in-person group intervention to telehealth and 2) experience and perceived benefit of attending a virtual group from the perspective of the participants of our first two groups. Briefly, our findings demonstrate the strong need for technological support. Participants report positive experience regarding the convenience of attending the group from their home, the benefits of the assigned exercises, and the support they found from other group members. The COVID-19 pandemic has forced many to embrace the virtual option as they adapt to a new normal. There are undoubtedly hurdles to overcome, but there are also advantages to be leveraged.

